# Are Type 2 Diabetes Mellitus and Depression Part of a Common Clock Genes Network?

**DOI:** 10.5334/jcr.159

**Published:** 2018-04-18

**Authors:** Ramanujam Karthikeyan, David Warren Spence, Gregory M. Brown, Seithikurippu R. Pandi-Perumal

**Affiliations:** 1 Plot No.144, South 9th Street, Meenakshi Amman Nagar, Surya Nagar, Madurai 625007, Tamil Nadu, IN; 2 Dufferin Street, Toronto, ON M6K 2B4, CA; 3 Department of Psychiatry, Centre for Addiction and Mental Health, University of Toronto, Toronto, ON, M5T 1R8, CA; 4 Somnogen Canada Inc, College Street, Toronto, ON, CA

**Keywords:** Circadian rhythm, Clock genes, T2DM, depression, diabetes

## Abstract

In recent years, there has been an increased prevalence of type 2 diabetes mellitus (T2DM) and depression across the world. This growing public health problem has produced an increasing socioeconomic burden to the populations of all affected countries. Despite an awareness by public health officials and medical researchers of the costs associated with these diseases, there still remain many aspects of how they develop that are not understood. In this article, we propose that the circadian clock could be a factor that coordinates both the neurobehavioral and metabolic processes that underlie depression and T2DM. We propose further that this perspective, one which emphasizes the regulatory effects of clock gene activity, may provide insights into how T2DM and depression interact with one another, and may thus open a new pathway for managing and treating these disorders.

Type 2 diabetes mellitus (T2DM) and depression are both significant public health problems whose worldwide prevalence is increasing [1]. Recent surveys show that diabetes affects about 425 million people or about 8.8% of the global population, thus making it one of the most common of all non-communicable diseases [2]. Additionally, about 300 million people globally report having gone through depressive episodes, and nearly 800,000 people end their lives as a result of suicidal mood states [3]. In 2017, $727 billion was spent on health care expenditures for diabetic patients aged between 20 and 79 years. Additionally, in the most recent year surveyed (2017), 4 million people among the global population died due to complications related to diabetes [2].

## The prevalence of depression and type 2 diabetes mellitus is underappreciated

The bidirectional relationship between diabetes and depression is well established [1,4]. T2DM is known to cause depression, and depression can act as an important risk factor for T2DM. These relationships can complicate and increase the challenge of managing these disorders. Further, evidence-based on epidemiological studies has shown that the occurrence of depression among patients with T2DM is nearly twice as great as that in the general population [4]. Additionally, the incidence of diabetes among patients with depression is 14% greater than that of the general populace [4]. These two observations provide indirect support for the suggestion that the two conditions often thought to be separate and independent entities may have reciprocal or mutually facilitating effects, and further, that there may be a common factor which underlies their pathogenesis. A greater understanding of the relationship between T2DM and depression could contribute significantly to the development of strategies for their treatment. In this article, we consider the associations which exist between clock gene activity and the occurrence of these two disorders. We further consider the practical implications of these associations.

The complexity of the factors that contribute to the development of depression, and indeed of the high prevalence of depression, is generally not appreciated by the public, nor have steps been taken in the educational sector, nor in the form of governmental policies, to increase awareness of the health impact of depression. Further, considerable work remains to be done to assist depressed individuals with more care, support, and service. A similar situation exists with respect to diabetes. The contribution of lifestyle factors to the development of diabetes is well known [5], however, the high incidence of depression among diabetic patients, and, further, the contribution of depression as a predisposing factor in T2DM, is less well-appreciated. Additionally, the mental health difficulties associated with diabetes have received far less attention than efforts to deal with T2DM’s linkage to, e.g., sedentary lifestyle factors and diet [6].

## Evidence pointing to the importance of clock genes in the development of type 2 diabetes mellitus and depression

An accumulating amount of studies has shown that the circadian clock system is involved in regulating both glucose metabolism [7,8,9,10,11,12] and mood behavior [13,14,15,16,17]. An argument has been made that circadian clock gene activity produces a regulatory network which, when it is functioning normally, maintains the natural rhythms that underlie optimal health. However, when the rhythms of this network are interfered with, through, e.g., poor sleeping habits or disrupted sleep, certain pathological conditions, such as diabetes and depression, may develop [18]. An assumption which has traditionally guided clinical practice and research is that poor nutrition and sedentary lifestyles are primary contributors to conditions such as T2DM, and are associated, at least in a correlative way, with depression. The relatively new circadian model of depression points strongly however to the possibility that disturbed sleep may be a major influence in the development of mood disorders such as depression [14,19] and indirectly, through its weakening of immune system activities, in diabetes [20,21]. These sleep-associated factors include dysregulation of the immune system, prolonged sleep perturbations and sleep deprivation [1]. Importantly, all these disorders have a bidirectional relationship with the circadian clock. Misalignment of circadian clock leads to insulin resistance [10,12] as well as to abnormal neurochemical changes involved in the regulation of mood [13,19,22,23]. Although the key roles played by the circadian timekeeping system in insulin synthesis and sensitivity is well known, studies focusing on the metabolic aspects of sleep and circadian rhythms have only appeared in recent decades [7]. Rhythmic changes, also regulated by circadian clock functioning, are observed in metabolic processes involved in glucose homeostasis; these include changes in circulating plasma glucose levels, insulin synthesis and sensitivity, food intake, adipogenesis, immune responses, and genomic expression in both liver and pancreas [24,25]. Master clock activities, as well as peripheral clock activity in liver and pancreas, rhythms of food intake, and other circadian activities, such as the release of melatonin and cortisol, profoundly influence the machinery associated with glucose homeostasis [26]. Current evidence shows that shift workers, whose circadian alignment may be adversely affected for as much as several years after they terminate their nighttime work schedules, also have an increased prevalence of diabetes [27]. A recent study of the effects of nighttime shift work has demonstrated that disrupted circadian clock functioning affects glucose tolerance by interfering with functional properties of the pancreatic cell during the biological evening [28]. Reduced levels of melatonin are observed in T2DM; further indicating that sub-optimal functioning of the circadian clock is responsible for aberrant glucose activity [29]. Insulin RNA and protein activity have been correlated with the release of clock gene products [11]. Further, evidence has been provided that the reduced expression of clock genes in patients with T2DM is associated with a dampening of circadian clock functioning [30]. Deletion of clock genes in mice has been shown to produce abnormal glucose metabolism resembling T2DM [8,9,10,12]. The experimental disruption of circadian rhythm activity through clock gene deletion was found to increase insulin resistance in mice due to the absence of the clock component [12]. The contribution of clock gene activity to the elevated insulin resistance was subsequently proven by the restoration of insulin resistance following the substitution of the clock gene product by its analogue [12].

Temporal adaptation of the nervous system and its components is essential for maintaining health in all mammals. An increasing amount of evidence has now been provided that circadian disruption may be an important cause of depressed mood [13,14,17,22]. It is known that in conditions such as seasonal affective disorder (SAD), in which depressed mood is associated with sunlight restriction and indoor confinement during the winter months, that circadian disruption and melatonin concentrations may be principal mediating factors [31]. Sleep quality influences neurobehavioral activities which prevail during the day time [32]. Considerable evidence has shown for instance that lack of sleep or sleep disruption not only alter mood, but can produce impulsivity, judgment errors, and impaired motor coordination [33]. Similarly, molecular activities which correlate with the regulation of mood, including the functioning of the hypothalamic-pituitary-adrenal (HPA) axis, sleep, and nervous system activity, all exhibit 24 h periodicities [17]. In addition, monoamines, endogenous opioids, and metabolic peptides demonstrate rhythmic patterns in their functioning [17]. Consistent with this evidence have been the findings of post-mortem investigations of the brains of patients who had suffered from major depressive disorder, and which, when compared to the brains of healthy subjects, exhibited reduced levels of clock gene expression due to shifted peak timing [34]. A weak association between the phase relationship of the circadian clock and environmental zeitgebers has been hypothesized to interfere with brain-regulated behavior [35,36]. Evidence from clinical studies is in line with these post-mortem findings. It has been shown for instance that disruption of circadian functioning is frequently correlated with depressive symptoms, and, conversely, that a common overt symptom of depression is sleep disturbance [13,15,19,22,23]. Additionally, it has been shown that depressive symptoms can be reduced by restricting the sleep of patients who exhibit simultaneously the symptoms of depressed mood and disturbed sleep [37,38,39]. All these findings support the view that the biological clock is strongly associated with normal and abnormal mood states of individuals, and, further, that this association may be mediated by clock gene activity, including regulation of carbohydrate homeostasis [24,25] and neurobehavioral processes [17]. This evidence supports the conclusion that disruption of circadian clock activity could be a major contributor to both diabetes and depression, and additionally that factors that interfere with this activity, such as disturbed sleep, may be a common denominator in the underlying pathogenesis of these debilitating disorders [18]. It is suggested here that greater efforts must be undertaken to develop awareness among researchers and health professionals regarding the important contribution of sleep hygiene to circadian clock activity, and, ultimately, to illnesses which depend on the maintenance of its normal functioning.

## Lifestyle effects on clock functions

Modern unhealthy lifestyles contribute in numerous ways to circadian disruption. The pressures of contemporary living are often accompanied by a lack of adequate physical activity, chronic sleep insufficiency, inappropriate sleep/wake schedules, exposure to artificial lighting at unusual times and diminished exposure to natural sunlight (duration and timing), occupational stressors (e.g. shift work, late night work), jet lag, poor eating habits (e.g. skipping breakfast, inconsistent timing of food intake), all of which can potentially disrupt the circadian clock and thus increase the risk of metabolic [24,25] and mental pathologies [17]. This is evident in the many health-related changes which have occurred in countries such as India, following the adoption of increasingly non-traditional and globalized social trends since the early nineteen-nineties. In 1990, the population incidence of diabetes was 15 million, a figure which doubled in 2010 and then doubled again by 2013 [40]. We believe that an argument could be made that the increasing complexity and pressures of modern “24/7” lifestyles are disruptive to normal biological clock functioning and that this phenomenon, in turn, has contributed to the ever-growing prevalence of mental illness in recent decades [17,41].

## Chronotherapeutic management of diabetes and depression

Clock genes affect neurobehavioral activities as well as the balance of glucose concentration in all cells. The broad-ranging influence of clock genes, with their functional role as a peripheral oscillator within neurons [42,43], brain [34], liver [44], pancreas [10], stomach [45,46] as well as their other molecular and cellular activities, provide compelling support for the hypothesis that they promote the development of both diabetes and depression. This point of view has led to the recent introduction of innovative treatments in which the timing of various therapies is carefully controlled. This treatment system, known as chronotherapeutics, is being increasingly used in clinical settings for treating depressed patients as well as those with a variety of sleep disorders. It has been shown for instance that resynchronization of environmental time cues with the circadian clock can improve the mood states of depressed patients, as well as those with other psychiatric disorders [47]. Light therapy [48,49], sleep deprivation [39,49], and social rhythm therapy [50] are some of the chronobiological therapies which are applied to enhance the mood of depressed patients. The various applications of chronotherapy have in common the goal of targeting clock genes and seeking to reset the clock in the suprachiasmatic nucleus, thus altering their functioning by abruptly changing the timing of sleep schedules of affected patients. This goal is achieved by the strategic scheduling of “zeitgebers” (environmental timekeepers) that occur in patients’ daily routines. Although chronotherapy is still a relatively new treatment approach and one whose application to conditions such as diabetes remains exploratory, it continues to attract a growing amount of interest among health professionals. This approach, which has been demonstrated to alter the expression of clock genes and, subsequently, to restore circadian rhythms to normal functioning, has now shown its effectiveness in enhancing the mood of depressed patients [16,51]. This innovative therapeutic strategy thus represents a potentially valuable non-drug alternative for treating mood disorders. Additionally, it may show promise for treating a broader range of conditions, including diabetes [25]. It is known for instance that melatonin can mediate the function of insulin [52] and, further, that administration of melatonin improves the function of insulin responsiveness [53].

In terms of basic research, many questions still remain about how clock genes exert control over mood expression and glycaemic functioning [1]. The basic mechanism appears to involve the activity of clock genes via circadian regulation of the sleep/wake cycle and by extension the control of melatonin synthesis. These activities, in turn, can dictate both the molecular and cellular processes associated with glucose metabolism and with the expression of mood. To characterize the association of how circadian clock disruption promotes the development of both depression and diabetes, more basic and clinical studies are needed. In particular, studies of the pathogenesis, morbidities, effects of pharmacological agents, and inter-individual differences in both disorders are needed [1]. Investigations of the chronic effects of circadian system and how its multiple components interact with each other to regulate insulin synthesis and psychological mood may provide new insights into the treatment of these important pathologies.

Considerable work remains to be done to develop further applied therapy programs for treating mood disorders and diabetes. Some efforts have been undertaken in this regard in a limited number of hospitals, however, a broad-ranging adoption of these newer treatment strategies into health institutions has not occurred, nor has the advocacy of this approach been incorporated into government policy in developed countries. Taken together, the findings cited above support advocacy of chronotherapy use not simply for clinical treatment but also as a common sense strategy that the general public may use for daily health maintenance. It is suggested that public health programs should emphasize practical steps that can be taken to ensure optimal functioning of the circadian clock. These could include recommendations that individuals receive adequate but not excessive amounts of sunlight exposure, that they schedule a reasonable amount of daily physical activity, and that they maintain a regular sleep/wake schedule. When adopted as part of one’s daily routine, these activities can significantly contribute to support normal energy balance and proper functioning of cell processes which are vital for sustaining optimal health.

## Agenda for the future and work that remains to be done

In view of the continuing personal and public health costs involved, there exists an urgent need to elucidate the pathogenic origin and treatment of depression and diabetes. Additionally, a continuing focus should be maintained on the fact that patient, family, and caretaker education is a pivotal component in the management of these disorders. Hence a multi-stakeholder perspective is necessary to analyze and meaningfully apply the results of basic research. The development of animal model studies as well as human clinical trials regarding the application of chronotherapeutic interventions may provide fresh insights into the inter-connectedness of circadian clock activity, mood behavior and glucose metabolism [24,25]. In summary, therefore, we advocate the adoption of a conceptual framework which emphasizes the importance of chronobiological effects in human health and disease. We believe that this is essential for enabling the global prevention, management, and treatment of T2DM and depression. It is suggested that this approach, one which emphasizes the key role of circadian clock activity, represents a relatively unexplored pathway for treating and reducing the suffering caused by depression and diabetes.

## References

[1] Holt, RI, De Groot, M, Lucki, I, Hunter, CM, Sartorius, N and Golden, SH. NIDDK international conference report on diabetes and depression: current understanding and future directions. Diabetes Care. 2014 8 1; 37(8): 2067–77. DOI: 10.2337/dc13-2134 25061135 PMC4113168

[2] International Diabetes Federation. IDF atlas. 2017; Eighth edition http://www.diabetesatlas.org/resources/2017-atlas.html [Accessed on February 2, 2018].

[3] World Health Organization. 2017; http://www.who.int/mediacentre/factsheets/fs369/en/ [Updated February, 2017, Accessed on February 5, 2018].

[4] Anderson, RJ, Freedland, KE, Clouse, RE and Lustman, PJ. The prevalence of comorbid depression in adults with diabetes: a meta-analysis. Diabetes Care. 2001 6 1; 24(6): 1069–78. DOI: 10.2337/diacare.24.6.1069 11375373

[5] Krug, EG. Trends in diabetes: sounding the alarm. The Lancet. 2016 4 9; 387(10027): 1485–6. DOI: 10.1016/S0140-6736(16)30163-5 27061675

[6] Macdonald, GC and Campbell, LV. Mental illness: the forgotten burden on diabetes populations? The Lancet. 2016 8 6; 388(10044): 561 DOI: 10.1016/S0140-6736(16)31213-2 27511779

[7] Van Cauter, E, Polonsky, KS and Scheen, AJ. Roles of circadian rhythmicity and sleep in human glucose regulation. Endocrine reviews. 1997 10 1; 18(5): 716–38. DOI: 10.1210/edrv.18.5.0317 9331550

[8] Rudic, RD, McNamara, P, Curtis, AM, Boston, RC, Panda, S, Hogenesch, JB and FitzGerald, GA. BMAL1 and CLOCK, two essential components of the circadian clock, are involved in glucose homeostasis. PLoS biology. 2004 11 2; 2(11): e377 DOI: 10.1371/journal.pbio.0020377 15523558 PMC524471

[9] Turek, FW, Joshu, C, Kohsaka, A, Lin, E, Ivanova, G, McDearmon, E, Laposky, A, Losee-Olson, S, Easton, A, Jensen, DR and Eckel, RH. Obesity and metabolic syndrome in circadian Clock mutant mice. Science. 2005 5 13; 308(5724): 1043–5. DOI: 10.1126/science.1108750 15845877 PMC3764501

[10] Marcheva, B, Ramsey, KM, Buhr, ED, Kobayashi, Y, Su, H, Ko, CH, Ivanova, G, Omura, C, Mo, S, Vitaterna, MH and Lopez, JP. Disruption of the clock components CLOCK and BMAL1 leads to hypoinsulinemia and diabetes. Nature. 2010 7; 466(7306): 627 DOI: 10.1038/nature09253 20562852 PMC2920067

[11] Stamenkovic, JA, Olsson, AH, Nagorny, CL, Malmgren, S, Dekker-Nitert, M, Ling, C and Mulder, H. Regulation of core clock genes in human islets. Metabolism-Clinical and Experimental. 2012 7 1; 61(7): 978–85. DOI: 10.1016/j.metabol.2011.11.013 22304835

[12] Shi, SQ, Ansari, TS, McGuinness, OP, Wasserman, DH and Johnson, CH. Circadian disruption leads to insulin resistance and obesity. Current Biology. 2013 3 4; 23(5): 372–81. DOI: 10.1016/j.cub.2013.01.048 23434278 PMC3595381

[13] Ehlers, CL, Frank, E and Kupfer, DJ. Social zeitgebers and biological rhythms: a unified approach to understanding the etiology of depression. Archives of general psychiatry. 1988 10 1; 45(10): 948–52. DOI: 10.1001/archpsyc.1988.01800340076012 3048226

[14] Germain, A and Kupfer, DJ. Circadian rhythm disturbances in depression. Human Psychopharmacology: Clinical and Experimental. 2008 10 1; 23(7): 571–85. DOI: 10.1002/hup.964 18680211 PMC2612129

[15] Hasler, BP, Buysse, DJ, Kupfer, DJ and Germain, A. Phase relationships between core body temperature, melatonin, and sleep are associated with depression severity: further evidence for circadian misalignment in non-seasonal depression. Psychiatry research. 2010 6 30; 178(1): 205–7. DOI: 10.1016/j.psychres.2010.04.027 20471106 PMC2914120

[16] Bunney, BG and Bunney, WE. Rapid-acting antidepressant strategies: mechanisms of action. International Journal of Neuropsychopharmacology. 2012 6 1; 15(5): 695–713. DOI: 10.1017/S1461145711000927 21733282

[17] McClung, CA. How might circadian rhythms control mood? Let me count the ways… Biological psychiatry. 2013 8 15; 74(4): 242–9. DOI: 10.1016/j.biopsych.2013.02.019 23558300 PMC3725187

[18] Moulton, CD, Pickup, JC and Ismail, K. The link between depression and diabetes: the search for shared mechanisms. The Lancet Diabetes & Endocrinology. 2015 6 1; 3(6): 461–71. DOI: 10.1016/S2213-8587(15)00134-5 25995124

[19] Borbély, AA. A two process model of sleep regulation. Hum neurobiol. 1982 5; 1(3): 195–204. PMID: 7185793. 7185792

[20] Shu, CJ, Benoist, C and Mathis, D. The immune system’s involvement in obesity-driven type 2 diabetes In. Seminars in immunology. 2012 12 1; 24(6): 436–442. Academic Press DOI: 10.1016/j.smim.2012.12.001 23333525 PMC3582811

[21] Scheiermann, C, Kunisaki, Y and Frenette, PS. Circadian control of the immune system. Nature Reviews Immunology. 2013 3; 13(3): 190 DOI: 10.1038/nri3386 PMC4090048 23391992

[22] Lewy, AJ, Sack, RL, Singer, CM, Whate, DM and Hoban, TM. Winter depression and the phase-shift hypothesis for bright light’s therapeutic effects: history, theory, and experimental evidence. Journal of Biological Rhythms. 1988 6; 3(2): 121–34. DOI: 10.1177/074873048800300203 2979635

[23] Lewy, AJ, Rough, JN, Songer, JB, Mishra, N, Yuhas, K and Emens, JS. The phase shift hypothesis for the circadian component of winter depression. Dialogues in clinical neuroscience. 2007 9; 9(3): 291. PMID: 17969866. 17969866 10.31887/DCNS.2007.9.3/alewy PMC3202495

[24] Kalsbeek, A, la Fleur, S and Fliers, E. Circadian control of glucose metabolism. Molecular Metabolism. 2014 7 1; 3(4): 372–83. DOI: 10.1016/j.molmet.2014.03.002 24944897 PMC4060304

[25] Karthikeyan, R, Marimuthu, G, Spence, DW, Pandi-Perumal, SR, BaHammam, AS, Brown, GM and Cardinali, DP. Should we listen to our clock to prevent type 2 diabetes mellitus? Diabetes research and clinical practice. 2014 11 1; 106(2): 182–90. DOI: 10.1016/j.diabres.2014.07.029 25172521

[26] Hampton, SM and Johnston, JD. Probing the diurnal regulation of glycemic control. Journal of Diabetes and its Complications. 2014 11 1; 28(6): 751–2. DOI: 10.1016/j.jdiacomp.2014.07.008 25161099

[27] Szosland, D. Shift work and metabolic syndrome, diabetes mellitus and ischaemic heart disease. International journal of occupational medicine and environmental health. 2010 1 1; 23(3): 287–91. DOI: 10.2478/v10001-010-0032-5 20934953

[28] Morris, CJ, Yang, JN, Garcia, JI, Myers, S, Bozzi, I, Wang, W, Buxton, OM, Shea, SA and Scheer, FA. Endogenous circadian system and circadian misalignment impact glucose tolerance via separate mechanisms in humans. Proceedings of the National Academy of Sciences 2015 Apr 28; 112(17): E2225–34. DOI: 10.1073/pnas.1418955112 PMC4418873 25870289

[29] McMullan, CJ, Schernhammer, ES, Rimm, EB, Hu, FB and Forman, JP. Melatonin secretion and the incidence of type 2 diabetes. Jama. 2013 4 3; 309(13): 1388–96. DOI: 10.1001/jama.2013.2710 23549584 PMC3804914

[30] Ando, H, Takamura, T, Matsuzawa-Nagata, N, Shima, KR, Eto, T, Misu, H, Shiramoto, M, Tsuru, T, Irie S, Fujimura, A and Kaneko, S. Clock gene expression in peripheral leucocytes of patients with type 2 diabetes. Diabetologia. 2009 2 1; 52(2): 329–35. DOI: 10.1007/s00125-008-1194-6 18974966

[31] Pandi-Perumal, SR, Srinivasan, V, Maestroni, GJ, Cardinali, DP, Poeggeler, B and Hardeland, R. Melatonin. Nature’s most versatile biological signal? The FEBS journal. 2006 7 1; 273(13): 2813–38. DOI: 10.1111/j.1742-4658.2006.05322.x 16817850

[32] Harvey, AG. Sleep and circadian rhythms in bipolar disorder: seeking synchrony, harmony, and regulation. American journal of psychiatry. 2008 7; 165(7): 820–9. DOI: 10.1176/appi.ajp.2008.08010098 18519522

[33] Foster, RG and Wulff, K. The rhythm of rest and excess. Nature Reviews Neuroscience. 2005 5; 6(5): 407 DOI: 10.1038/nrn1670 15861183

[34] Li, JZ, Bunney, BG, Meng, F, Hagenauer, MH, Walsh, DM, Vawter, MP, Evans, SJ, Choudary, PV, Cartagena, P, Barchas, JD and Schatzberg, AF. Circadian patterns of gene expression in the human brain and disruption in major depressive disorder. Proceedings of the National Academy of Sciences 2013 Jun 11; 110(24): 9950–5. DOI: 10.1073/pnas.1305814110 PMC3683716 23671070

[35] Souêtre, E, Salvati, E, Belugou, JL, Pringuey, D, Candito, M, Krebs, B, Ardisson, JL and Darcourt, G. Circadian rhythms in depression and recovery: evidence for blunted amplitude as the main chronobiological abnormality. Psychiatry research. 1989 6 1; 28(3): 263–78. DOI: 10.1016/0165-1781(89)90207-2 2762432

[36] Wirz-Justice, A. Chronobiology and psychiatry. Sleep medicine reviews. 2007 12 1; 11(6): 423–7. DOI: 10.1016/j.smrv.2007.08.003 18021941

[37] Landsness, EC, Goldstein, MR, Peterson, MJ, Tononi, G and Benca, RM. Antidepressant effects of selective slow wave sleep deprivation in major depression: a high-density EEG investigation. Journal of psychiatric research. 2011 8 1; 45(8): 1019–26. PMID: 21397252 DOI: 10.1016/j.jpsychires.2011.02.003 21397252 PMC3119746

[38] Szuba, MP, Baxter, LR, Fairbanks, LA, Guze, BH and Schwartz, JM. Effects of partial sleep deprivation on the diurnal variation of mood and motor activity in major depression. Biological Psychiatry. 1991 10 15; 30(8): 817–29. DOI: 10.1016/0006-3223(91)90237-G 1751624

[39] Wirz-Justice, A and Van den Hoofdakker, RH. Sleep deprivation in depression: what do we know, where do we go? Biological psychiatry. 1999 8 15; 46(4): 445–53. DOI: 10.1016/S0006-3223(99)00125-0 10459393

[40] Shetty, P. India’s diabetes time bomb. Nature. 2012 5 17; 485(7398): S14 DOI: 10.1038/485S14a 22616100

[41] Vigo, D, Thornicroft, G and Atun, R. Estimating the true global burden of mental illness. The Lancet Psychiatry. 2016 2 1; 3(2): 171–8. DOI: 10.1016/S2215-0366(15)00505-2 26851330

[42] Helfrich-Förster, C. The period clock gene is expressed in central nervous system neurons which also produce a neuropeptide that reveals the projections of circadian pacemaker cells within the brain of Drosophila melanogaster. Proceedings of the National Academy of Sciences 1995 Jan 17; 92(2): 612–6. DOI: 10.1073/pnas.92.2.612 PMC42792 7831339

[43] Rath, MF, Rovsing, L and Møller, M. Circadian oscillators in the mouse brain: molecular clock components in the neocortex and cerebellar cortex. Cell and tissue research. 2014 9 1; 357(3): 743–55. DOI: 10.1007/s00441-014-1878-9 24842045

[44] Schmutz, I, Albrecht, U and Ripperger, JA. The role of clock genes and rhythmicity in the liver. Molecular and cellular endocrinology. 2012 2 5; 349(1): 38–44. DOI: 10.1016/j.mce.2011.05.007 21664421

[45] Hoogerwerf, WA, Hellmich, HL, Cornélissen, G, Halberg, F, Shahinian, VB, Bostwick, J, Savidge, TC and Cassone, VM. Clock gene expression in the murine gastrointestinal tract: endogenous rhythmicity and effects of a feeding regimen. Gastroenterology. 2007 10 1; 133(4): 1250–60. DOI: 10.1053/j.gastro.2007.07.009 17919497

[46] Kentish, SJ, Frisby, CL, Kennaway, DJ, Wittert, GA and Page, AJ. Circadian variation in gastric vagal afferent mechanosensitivity. Journal of Neuroscience. 2013 12 4; 33(49): 19238–42. DOI: 10.1523/JNEUROSCI.3846-13.2013 24305819 PMC6618780

[47] Wirz-Justice, A, Bromundt, V and Cajochen, C. Circadian disruption and psychiatric disorders: the importance of entrainment. Sleep Medicine Clinics. 2009 6 1; 4(2): 273–84. DOI: 10.1016/j.jsmc.2009.01.008

[48] Rosenthal, NE, Sack, DA, Gillin, JC, Lewy, AJ, Goodwin, FK, Davenport, Y, Mueller, PS, Newsome, DA and Wehr, TA. Seasonal affective disorder: a description of the syndrome and preliminary findings with light therapy. Archives of general psychiatry. 1984 1 1; 41(1): 72–80. DOI: 10.1001/archpsyc.1984.01790120076010 6581756

[49] Wirz-Justice, A, Benedetti, F, Berger, M, Lam, RW, Martiny, K, Terman, M and Wu, JC. Chronotherapeutics (light and wake therapy) in affective disorders. Psychological medicine. 2005 7; 35(7): 939–44. DOI: 10.1017/S003329170500437X 16045060

[50] Ehlers, CL, Kupfer, DJ, Frank, E and Monk, TH. Biological rhythms and depression: the role of zeitgebers and zeitstorers. Depression. 1993 1 1; 1(6): 285–93. DOI: 10.1002/depr.3050010602

[51] Dallaspezia, S, Suzuki, M and Benedetti, F. Chronobiological therapy for mood disorders. Current psychiatry reports. 2015 12 1; 17(12): 95 DOI: 10.1007/s11920-015-0633-6 26478195

[52] Peschke, E, Peschke, D, Hammer, T and Csernus, V. Influence of melatonin and serotonin on glucose-stimulated insulin release from perifused rat pancreatic islets in vitro. Journal of pineal research. 1997 10 1; 23(3): 156–63. DOI: 10.1111/j.1600-079X.1997.tb00349.x 9406987

[53] Cuesta, S, Kireev, R, García, C, Rancan, L, Vara, E and Tresguerres, JA. Melatonin can improve insulin resistance and aging-induced pancreas alterations in senescence-accelerated prone male mice (SAMP8). Age. 2013 6 1; 35(3): 659–71. DOI: 10.1007/s11357-012-9397-7 22411259 PMC3636397

